# Genetic adaptations of the plateau zokor in high-elevation burrows

**DOI:** 10.1038/srep17262

**Published:** 2015-11-25

**Authors:** Yong Shao, Jin-Xiu Li, Ri-Li Ge, Li Zhong, David M. Irwin, Robert W. Murphy, Ya-Ping Zhang

**Affiliations:** 1 State Key Laboratory of Genetic Resources and Evolution, Kunming Institute of Zoology, the Chinese Academy of Sciences, Kunming 650223, China; 2 Laboratory for Conservation and Utilization of Bio-resources, Yunnan University, Kunming 650091, China; 3 Key Laboratory for High Altitude Medicine of Ministry of Chinese Education and Research Center for High Altitude Medicine, Qinghai University, Xining 810001, China; 4 Department of Laboratory Medicine and Pathobiology, University of Toronto, Ontario, M5S 1A8, Canada; 5 Banting and Best Diabetes Centre, University of Toronto, Ontario, M5S 1A8, Canada; 6 Centre for Biodiversity and Conservation Biology, Royal Ontario Museum, 100 Queen’s Park, Toronto, Ont., M5S 2C6, Canada; 7 Kunming College of Life Science, University of the Chinese Academy of Sciences, Kunming 650204, China

## Abstract

The plateau zokor (Myospalax baileyi) spends its entire life underground in sealed burrows. Confronting limited oxygen and high carbon dioxide concentrations, and complete darkness, they epitomize a successful physiological adaptation. Here, we employ transcriptome sequencing to explore the genetic underpinnings of their adaptations to this unique habitat. Compared to *Rattus norvegicus*, genes belonging to GO categories related to energy metabolism (e.g. mitochondrion and fatty acid beta-oxidation) underwent accelerated evolution in the plateau zokor. Furthermore, the numbers of positively selected genes were significantly enriched in the gene categories involved in ATPase activity, blood vessel development and respiratory gaseous exchange, functional categories that are relevant to adaptation to high altitudes. Among the 787 genes with evidence of parallel evolution, and thus identified as candidate genes, several GO categories (e.g. response to hypoxia, oxygen homeostasis and erythrocyte homeostasis) are significantly enriched, are two genes, *EPAS1* and *AJUBA*, involved in the response to hypoxia, where the parallel evolved sites are at positions that are highly conserved in sequence alignments from multiple species. Thus, accelerated evolution of GO categories, positive selection and parallel evolution at the molecular level provide evidences to parse the genetic adaptations of the plateau zokor for living in high-elevation burrows.

The plateau zokor, Myospalax baileyi, is small subterranean rodent that inhabits the Qinghai-Tibet Plateau at elevations ranging from 2000 to 4200 m^1^. Zokors spend 85–90% of their lives in underground nests, which are usually greater than 2 m deep for females and approximately 1.5 m deep for males^2^. The depths of the burrows means that the zokor experiences even lower levels of oxygen than those found on the surface of the Qinghai-Tibet Plateau and higher levels of carbon dioxide^3^.

The plateau zokor exemplifies a successful adaptation to an extreme environmental condition. Compared to pikas (*Ochotona curzniae*) and Sprague-Dawley rats (*Rattus norvegicus*), the plateau zokor exhibit remarkably increased red blood corpuscle counts and contents of hemoglobin and myoglobin^3^,^4^,^5^,^6^. In contrast, they have markedly lower pulse rates^6^, decreased lactate dehydrogenase activity, and lower 2,3-bisphosphoglycerate-content in their blood compared with pikas, mice and rats that live in the same area^3^. The oxygen pressure in the arterial blood of the plateau zokor is about 1.5 times higher than that of pikas and rats, and about 0.36 and 0.26 times, respectively, greater in venous blood^3^. Partial pressure for carbon dioxide in arterial and venous blood of the plateau zokor is 1.5-fold and 2.0-fold higher than in rats and pikas, respectively^3^. The difference in oxygen saturation of arterial to venous blood was about 2 and 4.5-fold higher in plateau zokor than in pikas and rats, respectively^3^. However, oxygen saturation of the plateau zokor is nearby 5.7 and 9.3 times lower in venous blood than that of pikas and rats, respectively^3^. These observations suggest that the plateau zokor has a strong capability to acquire oxygen from their hypoxic-hypercapnic environment.

To elucidate adaptations, and their genetic bases, of animals to extreme environments is a primary mission of modern evolutionary biology^7^. Recently developed bioinformatics help us comparatively analyze massive amounts of data generated by next-generation sequencing technologies at differential scales (e.g. genomics, transcriptomics and proteomics) across diverse species^8^,^9^,^10^. High altitude environments, especially low temperature, low oxygen density and intense UV radiation, result in harsh physiological challenges for animals^11^,^12^. A series of studies suggested that genes involved in the hypoxia-inducing factor (HIF) signaling pathway play key roles in the plateau adaptation of mammals^13^,^14^,^15^,^16^. Here, we examined the plateau zokor, a small endothermic mammal that experiences dual stress from high altitude hypoxia and sealed burrow hypoxia, and thus may become an excellent model for studying adaptations to low oxygen density and hypercapnic concentrations. Revealing the genetic bases of these adaptations in the plateau zokor therefore should be helpful to develop a comprehensive understanding of plateau adaptations of whole endotherms.

The naked mole rat, *Heterocephalus glaber*, is another strictly subterranean rodent^17^. Like the plateau zokor, they also live in full darkness, at low oxygen and high carbon dioxide concentrations and possess extremely similar habitats and physiological characteristics^18^. Thus, the plateau zokor and the naked mole rat represent a pair of functionally convergent homoplasies (evolutionary analogues) in adapting to the hypoxic environment of sealed burrows^19^. Utilization of the draft genome of the naked mole-rat^18^ together with the transcriptomes of multiple tissues from the plateau zokor should help us deeply explore genetic adaptations allowing the survival of these rodent species to harsh subterranean environments.

## Results

### *De novo* assembly and annotation of transcriptomes

We attained about 272.45 million (25.01 Gb sequence data) raw reads for five tissues (brain, liver, kidney, skeletal muscle and retina) of a plateau zokor. About 256.10 million (19.67 Gb) high-quality reads remained after quality control. The transcriptomes of the five tissues (brain, liver, kidney, skeletal muscle and retina) were pooled to generate an improved *de novo* assembly. The contig N50 parameters of the *de novo* assembly of the pooled data were superior to the single tissue transcriptome *de novo* assemblies, resulting in the identification of 208,451 non-redundant transcripts with a contig N50 length of 2433 base pairs (Table 1). The transcripts were aligned to the NCBI Non-Redundant Protein Database (NR database) using Blastx program (E-value 1e^−5^), allowing the annotation of 73,116 transcripts, based on 40,528 NCBI non-redundant proteins, including 34,523 (~85.2 %) that mapped to 19,736 NCBI genes (Supplementary Dataset 1). NCBI gene2go database (ftp://ftp.ncbi.nih.gov/gene/DATA) was then utilized to retrieve the GO ids for each of the annotated genes. In summary, 56 GO terms, mainly covering biological GO categories at three ontologies levels (23 GO terms in Biological Process, 15 GO terms in Molecular Function and 18 GO terms in Cellular Component), were identified using WEGO^20^, a web tool for plotting GO annotations. Our analyses of GO terms generally represented the main biological GO classification and ensured the integrity of the downstream functional analyses of the candidate genes.

### Predicted orthologous genes

We identified 8,217 genes that were one-to-one orthologous gene pairs between the plateau zokor (M. baileyi), and each of rat (*R. norvegicus*), kangaroo rat (*Dipodomys ordii*), guinea pig (*Cavia porcellus*), naked mole-rat (*H. glaber*) and human (*Homo sapiens*). After removing low quality and short sequences, 8,020 high-confidence one2one genes were retained for the downstream analyses. Our final dataset, therefore, included six species and 8020 genes.

### Phylogenetic tree and divergence time

**Figure 1:**
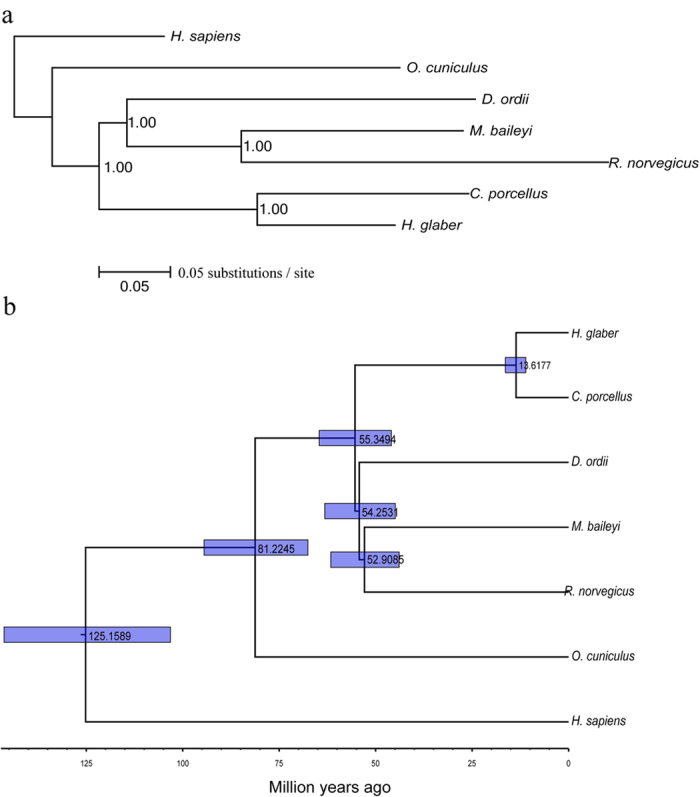
(**a**) Phylogenetic placement of the plateau zokor derived from an analysis of 4-fold degenerate sites from concatenated orthologous sequence by Mrbayes algorithm^21^. Substitutions/site and Bayesian posterior probabilities are shown. (**b**) The evaluation of the divergence times across seven mammalian species. The Bayesian relaxed molecular clock method was implemented using the BEAST v1.7.5 software^22^. We utilized an indirect estimate^18^ of the divergence time as the calibration point for the split of the ancestor of rabbit from the ancestor of mouse, rat and naked mole-rat. All nodal ages were indicated by medians and 95% HPD intervals (blue bars).

By integrating the 8020 single-copy orthologs among the six species (M. baileyi, *R. norvegicus*, *D. ordii*, *C. porcellus*, *H. glaber* and *H. sapiens*) and one-to-one orthologous gene pairs from *H. sapiens* and *Oryctolagus cuniculus*, downloaded from ENSEMBL 75 BIOMART database, a total of 7011 high-confidence single-copy orthologous gene pairs were obtained from the seven species (M. baileyi, *R. norvegicus*, *D. ordii*, *C. porcellus*, *H. glaber*, *O. cuniculus* and *H. sapiens*). After multiple sequence alignment (MSA) and quality trimming, 7011 one-to-one orthologous genes were concatenated into a single supergene to extract four-fold degenerate sites for construction of a phylogenetic tree using the Mrbayes_v3.0 software^21^. High Bayesian posterior probabilities in this constructed phylogenetic tree have well resolved the phylogenetic relationships between the plateau zokor and other rodent species (Fig. 1a). Based on this reliable phylogenetic tree, we estimated the divergence time of each node using BEAST v1.7.5^22^. As expected, the plateau zokor groups with the rat with an ancestor approximately ~52 million years ago, whereas the ancestor of the plateau zokor, rat and kangaroo rat diverged from the ancestor of the guinea pig and naked mole-rat approximately ~55 million years ago (Fig. 1b). Our estimated divergence time are similar to those of a previous report^23^.

### Evolutionary rate in plateau zokor

**Figure 2:**
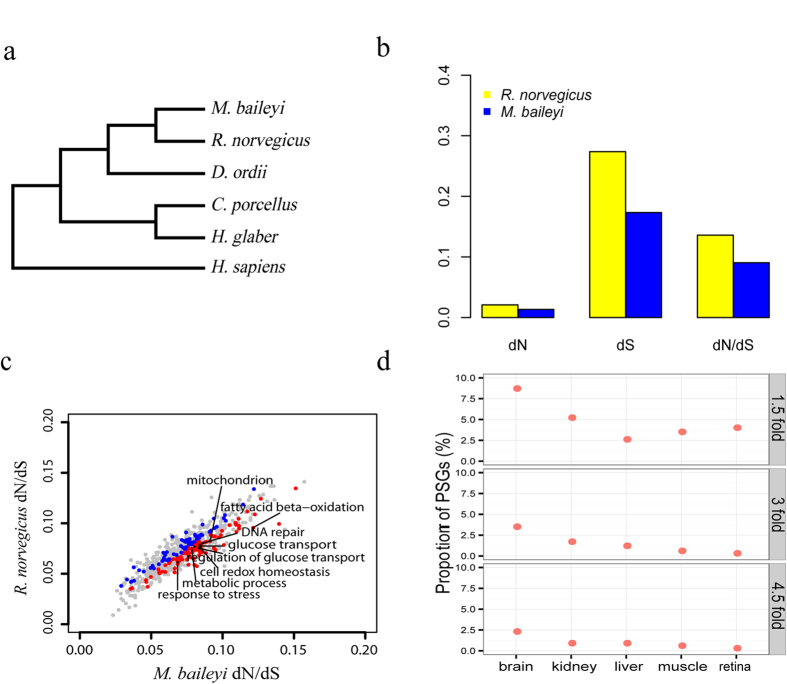
(**a**) Tree topology of six mammal species. This tree topology originated from our phylogenetic analyses among seven mammal species (*M. baileyi*, *R. norvegicus*, *D. ordii*, *C. porcellus*, *H. glaber*, *O. cuniculus* and *H. sapiens*). (**b**) Comparison of the evolutionary rates between the plateau zokor and rat lineages. Mean *d*_*N*_, *d*_*S*_, and *d*_*N*_*/d*_*S*_ ratio were originated in 7077 reliable orthologous gene pairs of both lineages are presented. (**c**) Comparisons of the *d*_*N*_*/d*_*S*_ ratios between the plateau zokor and rat lineages by GO functional categories. Blue and red dots represented categories with an elevated evolutionary rate along the rat and plateau zokor lineages, respectively. X-axis standed for the mean *d*_*N*_*/d*_*S*_ ratio of each GO functional category in the plateau zokor lineage and Y-axis standed for the mean *d*_*N*_*/d*_*S*_ ratio of each GO functional category in the rat lineage. (**d**) Percentage of tissue-specifc highest expressed positively selected genes in each tissue compared to the total number of positively selected genes in the plateau zokor lineage. The 1.5, 3 and 4.5 fold indicate the expression (log_2_ (fpkm+1)) of positively selected genes in a single tissue is greater than 1.5, 3 and 4.5 fold of any other tissue, respectively. The y-axis shows the percentage of the number of tissue-specifc highest expressed positively selected genes compared to the number of total positively selected genes in the plateau zokor lineage. The PSGs in the figure are the positively selected genes.

**Figure 3:**
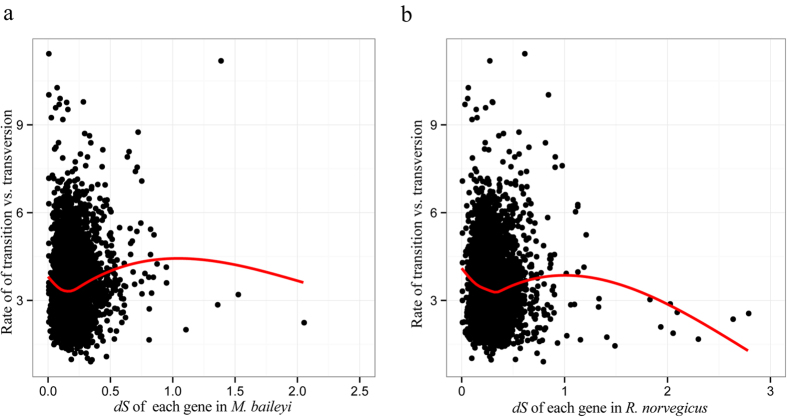
Saturation test of the one-to-one orthologous gene pairs. Each ratio (transition/transversion) of gene was calculated by the free-ratio model of PAML4^24^. (**a**) Plot of the ratio (transition/transversion) vs. *d*_*S*_in the plateau zokor lineage. (**b**) Plot of the ratio (transition/transversion) vs. *d*_*S*_ in rat lineage.

We utilized the free ratio model (M1) in PAML4^24^ to calculate the selective constraints acting on each of the orthologous genes using our accepted tree topology (Fig. 2a). Because outlier-genes with larger *d*_*N*_/*d*_*S*_ may generate deviations in evaluating the overall selective constraint in species, our dataset was filtered to remove genes with *d*_*N*_/*d*_*S*_ > 4, yielding a set of 7117 othologs shared by the plateau zokor and rat lineages. As saturation of substitutions influences the estimates of *d*_*N*_*/d*_*S*_, we used previously described methods^25^ to test for saturation and plotted the ratio of transitions and transversions to *d*_*S*_; these relationships were linear up to the *d*_*S*_ of 1, thus orthologues with *d*_*S*_ > 1 were excluded from further analyses (Fig. 3). After the saturation test, 7077 orthologous genes were remained, with these highly reliable genes used to evaluate the overall selective constraints in these two related rodent species. The low overall *d*_*N*_/*d*_*S*_ mean value in the plateau zokor (0.0904) and the rat (0.1360) demonstrates that the majority of genes experienced purifying selection on both lineages. Compared to the rat, the plateau zokor has a significantly lower mean *d*_*N*_/*d*_*S*_ value at the genomic level (Wilcoxon rank sum test, *P* < 2.2E–16) (Fig. 2b), suggesting that genes evolved a lower rate in the plateau zokor compared to rat since their split from a common ancestor. The mean d_*N*_/*d*_*S*_ value (0.1360) for the rat calculated in this study is very similar to that of a previous study (0.137)^26^, suggesting that our analysis is reliable.

### Accelerated evolution of genes in specific GO categories

Average *d*_*N*_*/d*_*S*_ values for genes in each GO category were calculated based on our reconstructed tree topology (Fig. 2a) using the free ratio model (M1) implemented in PAML4^24^. Among the Go categories, 95 harbor significantly higher average *d*_*N*_*/d*_*S*_ values in the plateau zokor lineage compared to the rat (*P* < 0.05, binomial test), with these categories including those involved in energy metabolism including glucose transport (GO:0015758, P = 6.58E–15), regulation of glucose transport (GO:0010827, P = 3.09E–11), mitochondrion (GO:0005739, P = 1.34E–06), fatty acid beta-oxidation (GO:0006635, P = 0.0004) (Fig. 2c and Supplementary Dataset 2). In contrast, the rat possessed only 65 categories exhibiting significantly higher *d*_*N*_*/d*_*S*_ values compared to the plateau zokor, which included those for collagen (GO:0005581, P = 3.22E–58) and protein polyubiquitination (GO:0000209, P = 1.50E–06).

### Positive selection

The improved branch-site model in PAML4^24^ was used to detect positively selected genes (PSGs) among the 8,020 orthologous genes along the plateau zokor branch using our reconstructed tree topology (Fig. 2a). A total of 346 PSGs (4.3%) were identified with signals of positive selection (Supplementary Dataset 3). G:Profiler^27^ enrichment analyses showed that the candidate PSGs were significantly enriched into categories that included response to stress (P = 4.28E–16), respiratory gaseous exchange (P = 9.31E–05), blood vessel development (P = 1.54E–03) and ATPase activity (P = 1.32E–04) (Supplementary Dataset 4). These categories appear to be biologically relevant to adaptation to high altitudes. According to previous studies, angiogenesis is largely initiated by the HIF pathway and is a crucial response to hypoxia^28^,^29^. Here, 12 PSGs (*ANGPTL3*, *PRCP*, *SEC24B*, *EPHA1*, *MCAM*, *KAT6A*, *MYH9*, *CYSLTR2*, *MYLK*, *SPINT1*, *PPAP2B* and *STRA6*) involved in blood vessel development were identified in the plateau zokor and they may represent adaptive responses to hypoxia. Since ATPase genes have a role in providing energy in conditions of low PO_2_, and show evidence of adaptation in the Tibetan antelope (*Pantholops hodgsonii*)^30^, another sympatric plateau species, PSGs associated with ATPase activity also may be adaptive to supply energy in the extremely hypoxic sealed burrow environment at high altitudes for the plateau zokor.

In particular, 5 PSGs (*SFTPA2*, *CSF2RB*, *MAPK8IP3*, *PASK* and *MTG2*) involved in respiratory gaseous exchange were over-represented. This category was not found to over-enriched in PSGs in studies of other plateau species, such as Tibetan antelope (*P. hodgsonii*)^30^, Tibetan boar (*Sus scrofa*)^31^ and yak (*Bos grunniens*)^16^, thus these genes may be specific to the plateau zokor and involved in the adaptive evolution of the respiratory system response to hypoxic-hypercapnic environment. In addition, tissue-specific analyses of the expression of PSGs showed the tendency that the brain hosted a higher proportion of PSGs with highest expression than any other tissue (kidney, retina, muscle and liver) (Fig. 2d).

### Convergent/parallel evolution

Adaptive evolution at the molecular level also can be studied by detecting convergent/parallel evolution at the amino acid sequence level^32^. Since both the plateau zokor and naked mole-rat live in sealed burrows and possess extremely similar phenotypic physical characteristics, we detected convergent/parallel evolved genes to reveal possible adaptive mechanisms on these two lineages. In summary, no convergently evolved genes were identified between the plateau zokor and naked mole-rat, however 787 candidate parallel evolved genes, including 700 having a single parallel amino acid change, 72 with two parallel amino acid substitutions and 15 having multiple (>2) parallel changes were identified on the two branches (Supplementary Dataset 5).

**Figure 4:**
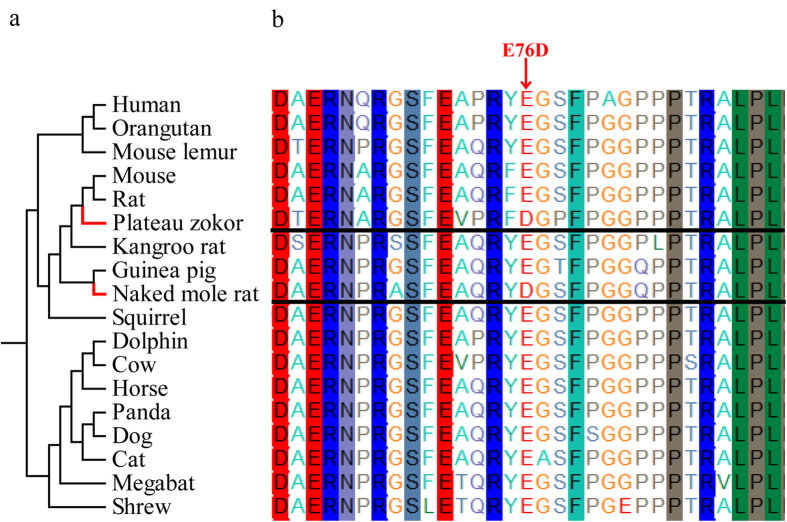
(**a**) Topology of the species tree. In this species tree, the two parallel evolving branches (plateau zokor and naked mole rat) were highlighted in red. (**b**) Multiple sequence alignments (MSAs) of the predicted coding sequences of *AJUBA* plateau zokor and other species. The MSAs were generated by PRANK^55^,^56^,^57^ with the aligned coding sequence translated into protein sequence using MEGA5^63^. In the protein sequences, the parallel evolved site (E76D) in the plateau zokor is indicated by the red arrow.

Our GO enrichment analyses of these candidate PEGs showed that they were significantly overrepresented in GO categories such as response to hypoxia (P = 3.27E–04), oxygen homeostasis (P = 5.85E–03), erythrocyte homeostasis (P = 3.25E–02), angiogenesis (P = 5.63E–08), blood vessel development (P = 1.69E–10), respiratory tube development (P = 8.75E–03) and ATPase activity (P = 6.79E–07) (Supplementary Dataset 6). Some of these GO categories are similar to the GO categories identified in the PSG analysis, suggesting that positively selected and parallel evolved genes exist in the same pathways (e.g. blood vessel development, respiratory tube development and ATPase activity) hinting that these genes related to energy metabolism and the development of the respiratory tube may contribute vital roles to adapting to the extremely high-elevation hypoxic burrow environment. However, in contrast, some significant GO categories (e.g. erythrocyte homeostasis, response to hypoxia and oxygen homeostasis) only were specifically enriched in the PEGs list and these specific GO categories possibly directly contribute to the burrow adaptation at high altitude because of their functional importance. Among these categories, 12 genes (*EPAS1*, *SLC29A1*, *MYO5B*, *AJUBA*, *TGFBR3*, *VASN*, *ACAA2*, *PINK1*, *SIRT1*, *SIRT2*, *SOD2* and *NARFL*) are involved in response to hypoxia; 5 genes (*EPAS1*, *DNASE2*, *ADAR*, *TGFBR3* and *SMAP1*) are involved in erythrocyte homeostasis and two genes (*SOD2* and *NARFL*) are involved in oxygen homeostasis. Since the response to hypoxia GO category is generally associated with high-elevation adaptation^15^,^33^, this suggests that their parallel evolved sites may not have appeared by random, but instead possess important functions, although the functions of these sites remain unclear. The gene *AJUBA* is an example, where an examination of homologous coding sequences in diverse mammals showed that the parallel evolved site (Glu76Asp) in the plateau zokor (is homologous to 86Glu site in human) occurs at an otherwise extremely conserved site in other species (Fig. 4).

### Protein-Protein Interaction (PPI) Network

**Figure 5:**
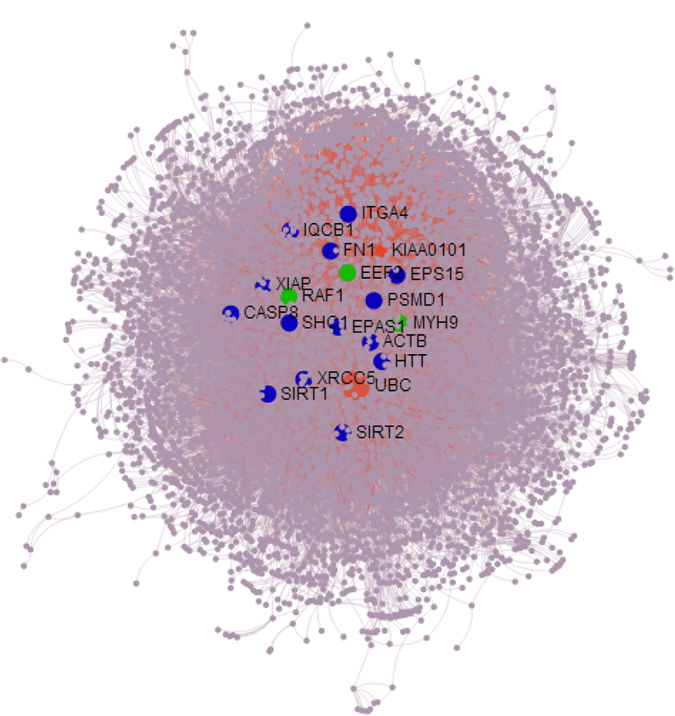
The PPI (Protein-Protein Interaction) network of the candidate genes. Candidate genes with more than 100 interactions (degree ≥ 100) are named and indicated by green dots for PSGs and blue dots for PEGs. PSGs are positively selected genes and PEGs are parallel evolved genes.

As mapping of candidate genes to protein-protein interaction (PPI) networks is essential to understand their biological functions, we combined the PSGs and PEGs, resulting in a total of 1,133 candidate genes, and mapped them to the PPI network database (InnateDB)^34^ to further explore their functional roles. Sub-networks of more than 5 nodes were retained and our analyses resulted in the identification of 935 seed proteins (queries) (82.5%) mapped to sub-networks consisting of 5,954 nodes (proteins) and 15,871 edges (protein-protein interactions) (Fig. 5). Of the 23 genes with the highest degree of interaction (> 100), 14 (*FN1*, *ITGA4*, *SIRT1*, *SHC1*, *ACTB*, *IQCB1*, *XRCC5*, *HTT*, *CASP8*, *XIAP*, *EPS15*, *PSMD1*, *EPAS1* and *SIRT2*) underwent parallel evolution, with 3 of these (*RAF1*, *EEF2 and MYH9*) also experiencing positive selection. Our results suggest that PSGs and PEGs may play central roles in maintaining these sub-networks.

## Discussion

The plateau zokor is a specialized species endemic to the Qinghai-Tibet Plateau^35^, which may have evolved a series of physiological strategies to counter the effects of hypoxia^3^ and can be regarded as an alternative evolved pattern to study adaptation to high elevation. Here, we report the transcriptomic data for a plateau zokor with a total of 20,8451 non-redundant transcripts from pooled multiple-tissue data with a Contig N50 attained length of 2,433 base pairs. The length of our sequences allowed the high-confident identification of orthologous genes, which could then be used to perform evolutionary analyses for plateau adaptation. Our analyses meaningfully contribute to the study of the genetic bases of adaptive evolution to high elevation in plateau mammalian species.

After diverged from their ancestor, compared to the rat lineage, overall the plateau zokor displayed a slower mean evolutionary rate in genomic level, although it possesses genes with more rapid evolution in GO categories related to energy metabolism. This result supports the hypothesis that highland adaptation of endothermic species mainly involves several features, including expanded gene families, elevated evolutionary rates and positive selection on genes involved in hypoxia and energy metabolism^16^,^30^,^36^. Significantly accelerated evolution of genes in energy metabolism also support the conclusion that the plateau zokor obtains most of its energy by aerobic oxidation instead of anaerobic glycolysis^37^. Adaptive evolution at the molecular level was well reflected in our screens for positive selection that identified genes involved adaptation^38^. In the plateau zokor lineage, genes involved in energy metabolism, such as ATPase activity experienced positive selection and thus we suggest that this category of genes may be relevant to adaptation to high altitudes. PSGs associated with blood vessel development were also over-represented and may contribute their function to hypoxic responses as angiogenesis is usually considered to be a target of the HIF pathway^28^,^29^. Several genes are of particular interest due to their functional implications. For example, *ANGPTL3* stimulates endothelial cell adhesion and migration via integrin and induces blood vessel formation *in vivo*^39^. This gene experienced positive selection suggesting an important function in angiogenesis. Another gene, *PRCP* (serine protease prolylcarboxypeptidase), might influence systemic blood pressure and vascular anticoagulation and contributes to cell proliferation and angiogenesis^40^, promoting the health or impair the repairs of blood vessels.

Our study also identified a potentially new GO category for plateau adaptation, respiratory gaseous exchange, as it was significantly enriched in our PSGs list. This category was not found in previous analyses for positive selection in other plateau species (yak (*B. grunniens*), Tibetan boar (*Sus scrofa*), Tibetan antelope (*P. hodgsonii*) and ground tit (*Parus humilis*))^16^,^30^,^31^,^36^, suggesting that it might be specific to the plateau zokor. For example, the candidate genes *SFTPA2* and *CSF2RB* are involved in surfactant structure and the regulation of surfactant homeostasis in the lung^41^,^42^. These genes may explain at molecular level why the plateau zokor possess a large capacity for lung gas diffusion. The number of PSGs with tissue-specific highest expression in brain is higher than in other tissues (e.g. liver, kidney, muscle and retina), suggesting that PSGs prefer to be highly expressed in the brain, since the brain might contribute more than other tissues to the response to hypoxia. We know for humans that the brain has high energy requirements, and consumes nearly 20% of the oxygen and 25% of the glucose of the entire body^43^. Thus, in the plateau zokor, the brain may similaily consume a high proportion of the energy and oxygen.

Adaptive evolution at the molecular level can also be studied by detecting convergent/parallel evolution at the amino acid sequence level^32^. Species living in similar ecological environments could be shaped by convergent evolution to form physiological or morphological similarities^44^. The naked mole-rat also lives in a strictly subterranean habitat that involves full darkness and low oxygen and high carbon dioxide concentrations^18^,^45^,^46^. Our GO functional analyses showed parallel evolved genes were significantly enriched in the following GO categories: response to hypoxia, ATPase activity and angiogenesis. These GO categories illustrate that in order to survive their shared harsh environment, the plateau zokor and naked mole-rat may experience convergent functional improvements in the supply of optimum oxygen levels by influencing the function of genes related to the hypoxia response, energy mechanism and angiogenesis. For example, the gene *EPAS1* encodes HIF-2α, which primarily regulates the production of erythropoietin (EPO). In turn, EPO is a key hormone that stimulates and regulates the production of erythrocytes^47^*. EPAS1* is widely reported as a candidate adapted gene for living at high elevations^13^,^15^,^48^. Additionally, analysis of Protein-Protein interaction (PPI) networks also demonstrates that *EPAS1* possesses strong interactions with other proteins. Taken together, *EPAS1* in the plateau zokor and naked mole-rat lineages exhibited parallel changes of amino acid sequences that are likely not due to random chance, but are an adaptive mechanism in response to decreased oxygen in their sealed environments. Another gene, *AJUBA*, is also involved in response to hypoxia and is a key regulator of the hypoxic response regulating the cellular and physiological changes to oxygen levels by controlling the degradation, and transcriptional activity of hypoxia-inducible transcriptional activity, of hypoxia-inducible transcription factors (HIFs)^49^. In particular, this gene hosts a highly conserved parallel evolved site (E76D) (identified in comparison of homologous sequences among multiple species) that suggests that it is also associated with highland adaptation, although the function of this particular amino acid substitution is unknown. Future functional experiments will be necessary to validate its importance. Thus, the plateau zokor exhibits multiple strategies to adapt to harsh highland environments. Although we did not directly examine the role of differentially expressed genes, due to the limitations of our sampling strategy, changes in gene expression likely also play important roles in the phenotypic evolution of this species^50^.

## Materials and Methods

Methods were carried out in accordance with approved guidelines.

### Sample collection and library preparation

The care and treatment of the plateau zokor comply with the guidelines for the National Care and Use of Animals approved by the National Animal Research Authority (P. R. China). Experimental protocols involving live animals were approved by the Ethics and Experimental Animal Committee of Kunming Institute of Zoology, Chinese Academy of Science, China (Approval ID: SYDW-2015012).

A plateau zokor individual was collected in Qinghai Province and after euthanasia, the brain, liver, kidney, skeletal muscle and retina tissue were quickly biopsied, placed in liquid nitrogen, and stored at −80 °C upon return to the laboratory. Total RNA from these tissues was extracted using TRIzol reagent (Invitrogen Corp., Carlsbad, CA). RNA purifications were performed using an RNeasy Mini Kit (Qiagen, Chatsworth, CA). Library constructions from the brain, liver and kidney of a plateau zokor (no replicates) followed the Illumina Genome Analyzer II RNA sample preparation kit (GA IIx, Illumina, Inc.) and Library preparation for skeletal muscle and retina (no replicates) were according to the Illumina Hiseq2000 RNA sample preparation kit (Illumina, San Diego, CA). All original data were deposited in the NCBI Sequence Read Archive database (Accession Number: SRP057676; Alias: PRJNA282349).

### *De novo* assembly and annotation of transcriptomes in plateau zokor

Sequencing adaptors used for cDNA library construction were trimmed using Cutadapt^51^ (version_1.2.1), which removed adapter sequences from the high-throughput sequencing reads. We employed Btrim64^52^ (version_0.1.0) to delete regions with average quality scores of less than 20 and impose a minimal length equal or great than 20 bp. High quality pooled paired end reads from multiple tissues were *de novo* assembled using Trinity^53^ (version_2013_08_14) by default parameters. Transcripts of pooled transcriptome were aligned to the NCBI Non-redundant protein database (NR) using BLASTX program (E-value 1E^−5^) to produce annotation results. NCBI gene2accession and gene2go databases (ftp://ftp.ncbi.nih.gov/gene/DATA) were then used, respectively, to retrieve gene ID and GO ID for each annotated gene. WEGO software^20^ (http://wego.genomics.org.cn/cgi-bin/wego/index.pl) was used to perform functional annotation analyses at three gene ontology levels (Biological Process, Molecular Function and Cellular Component).

### Prediction of open reading frames

Coding sequences of the plateau zokor were predicted based on the assumption that the longest open reading frame in the longest transcript per gene had a greatest chance of being a protein-coding region. GETORF in EMBOSS^54^ (version_6.4.0) was applied to obtain the nucleic sequences between the start and stop codons and limit the minimum size of a fragment to 120 bp. The transeq program in EMBOSS^54^ (version_6.4.0) was then used to obtain translated protein sequences.

### Identification of one-to-one orthologous genes

We used the longest protein sequences per gene to perform a best reciprocal hit (BRH) (E-value 1E^−5^) methods to identify one-to-one orthologous genes among six species: plateau zokor (M. baileyi), rat (*R. norvegicus*), kangaroo rat (*D. ordii*), guinea pig (*C. porcellus*), naked mole-rat (*H. glaber*), and human (*H. sapiens*). Protein sequences of the naked mole rat were downloaded from the naked mole rat database (http://mr.genomics.org.cn/page/species/index.jsp). Genomic protein sequences of the rat, kangaroo rat, guinea pig and human were downloaded from ENSEMBL 75 (http://www.ensembl.org/). Predicted orthologous genes were submitted to multiple alignments using PRANK^55^,^56^,^57^ (Parameters: −f = fasta -F -codon -noxml -notree -nopost). We aligned the longest ORFs for the longest transcript-pairs across the six species. Gblocks^58^,^59^ (version_0.91b; Parameters: −t = c −b3 = 1 −b4 = 6 −b5 = n) was employed to reduce the rate of false positive predictions by filtering out sequencing errors, incorrect alignments and no-orthologous regions based on codons. After trimming, we removed alignment lengths shorter than 10 bp to obtain one2one orthologous genes.

### Phylogenetic tree and divergence time

To establish phylogenetic relationship of the plateau zokor to other rodent mammals, one-to-one orthologous gene pairs between the human (*H. sapiens*) and rabbit (*O. cuniculus*) were downloaded from ENSEMBL 75 BIOMART database and added to dataset of one-to-one orthologous described above, generating one-to-one orthologous gene pairs for seven species. After multiple sequence alignments and trimming by the programs PRANK^55^,^56^,^57^ and Gblocks^58^,^59^, respectively, these single-copy orthologous genes were concatenated into one supergene and 4-fold degenerate sites were extracted and used to construct a phylogenetic tree. Modeltest_0.1^60^ was used to select the best substitute model and Mrbayes_v3.0^21^ was utilized to reconstruct the phylogenetic tree. Chain length was set to 10,000,000, with the first 1,000 samples treated as burned in, and the other parameters were set as defaults. Nodal ages within rodent mammals were evaluated, along with their 95% confidence intervals (CIs), from our 4-fold degenerate site data from the supergene sequence using the Bayesian relaxed molecular clock method implemented in BEAST v1.7.5^22^. Chain length was set to 10,000,000, with the first 1,000 samples treated as burned in. We utilized an indirect estimate^18^ of the divergence time as a calibration point with the split of the ancestor of rabbit from the ancestor of mouse, rat and naked mole-rat to estimate the internal nodal ages.

### Evolutionary rate and accelerated evolution of GO categories

Free ratio model (M1) (Parameters: model = 1, NSsites = 0, fix_omega = 0, omega = 1) in PAML4^24^ was used to calculate selective constraint of each orthologous gene using our constructed tree topology. Genes with *d*_*N*_/*d*_*S*_ >4 were first filtered and then a saturation test was performed to produce a final dataset to evaluate the overall selective constraints in the tested species. GO term information was downloaded from ENSEMBL (http://www.ensembl.org), and only those GO categories with more than 20 orthologs were included in our analyses. Lineage-specific mean *d*_*N*_/*d*_*S*_ values were estimated by concatenating alignments from all orthologs for each GO category. Relatively accelerated evolutionary GO terms were identified using a binomial test according to a previous study^26^.

### Positive selection analyses

Branch-site model (Parameters: Null hypothesis: model = 2, NSsites = 2, fix_omega = 1, omega = 1; Alternative hypothesis: model = 2, NSsites = 2, fix_omega = 0, omega = 1) in PAML4^24^ was used to detect positive selection in the one-to-one orthologous genes using our tree topology as the guide tree. Compared to other estimation models, the Branch-site model has the advantage of detecting positive selection that affects only a few sites on a pre-specified (foreground) branch of the species tree. The likelihood rate test (LRT) was used to detect positive selection on the foreground branch. PSGs were inferred only if their *P* values were less than 0.05. After identifying positively selected genes, the Bayes empirical Bayes (BEB) method was implemented to calculate posterior probabilities and to record positively selected sites. P-values of all PSGs also were normalized by FDR using Benjamini & Hochberg approach^61^. G:Profiler software^27^ was utilized to perform functional enrichment analyses for the PSGs by using ‘all known genes’ of human as the statistical background domain size. Additionally, we deduced that the variable trend of tissue-specific highest expressed PSGs in plateau lineage. Here, our analyses controlled the strict gradient thresholds with folds in expression level (>1.5fold, >3fold and >4.5fold) to ensure this trend more accurate. In brief, if log_2_ (fpkm + 1) of the PSGs in one tissue respectively was greater 1.5, 3 and 4 fold of the expressions of all other four tissues, then the PSG was regarded to be the tissue-specific highest expressed gene.

### Convergent/parallel evolution

Convergent/Parallel evolved changes were identified using the PAML4^24^ package to reconstruct the most likely ancestral states, and then a PERL program was used to calculate the number of parallel amino acid replacements for a specified pair of branches. If the posterior probability of the reconstructed ancestral amino acid site was more than 90%, then they were retained and their states were deemed as reliable. In addition, G:Profiler software^27^ was used to perform functional enrichment analyses for these PEGs utilizing ‘all known genes’ of human as the statistical background domain size.

### Protein-Protein Interaction (PPI) Network

We mapped the combined PSGs and PEGs candidate gene lists to the protein-protein interaction (PPI) network database (InnateDB)^34^. Sub-network topologies were obtained by NETWORKANALYST software^62^ (http://www.networkanalyst.ca/NetworkAnalyst). Sub-networks with node counts ≤5 were filtered and the statistics of total nodes, edges and seed proteins were obtained from the mapping overview.

## Additional Information

**How to cite this article**: Shao, Y. *et al.* Genetic adaptations of the plateau zokor in high-elevation burrows. *Sci. Rep.* **5**, 17262; doi: 10.1038/srep17262 (2015).

## Supplementary Material

Supplementary Dataset 1

Supplementary Dataset 2

Supplementary Dataset 3

Supplementary Dataset 4

Supplementary Dataset 5

Supplementary Dataset 6

## Figures and Tables

**Table 1:** The summary of the transcriptome data for a plateau zokor.

Tissue	Raw Reads Number	Clean Reads Number	Contigs Number	Contigs N50 (*bp)
Brain	60,294,120	57,366,679	66674	2371
Kidney	44,738,308	43,607,559	51306	2275
Liver	55,713,348	53,250,755	43874	1617
Muscle	67,915,990	62,924,369	99059	884
Retina	43,793,426	38,956,940	90362	812
Mixture	272,455,192	256,106,302	208451	2433

^*^bp = base pair.
